# Fear and Anger in Great Britain: Blame Assignment and Emotional Reactions to the Financial Crisis

**DOI:** 10.1007/s11109-013-9241-5

**Published:** 2013-07-09

**Authors:** Markus Wagner

**Affiliations:** Department of Methods in the Social Sciences, University of Vienna, Rathausstrasse 19/1/9, 1010 Vienna, Austria

**Keywords:** Accountability, Anger, Blame assignment, Economic perceptions, Emotional reactions, Fear

## Abstract

While we know that emotional reactions are important influences on political behavior, we know far less about the sources of these emotions. This paper studies the causes of fear and anger in reaction to a negative stimulus: the financial crisis. Anger should have been experienced among individuals who believed a specific actor was to blame for the crisis. Moreover, individuals should have been particularly angry if they blamed an actor who should be accountable to them, for example the national government. I test these expectations using a panel survey run in Britain between 2005 and 2010. This data shows that British citizens experienced anger if they held an actor responsible for the crisis. Moreover, they felt particularly angry if they held the Labour government (and to a lesser extent the European Union) responsible. These findings underline the importance of studying the causes of emotional reactions and show how these may be linked to common institutional distinctions between political systems.

**Electronic supplementary material:**

The online version of this article (doi:10.1007/s11109-013-9241-5) contains supplementary material, which is available to authorized users.

## Introduction

The emotions we experience have important consequences for our political behavior. Recent psychological and neuroscientific research has thus shown that our emotional reactions can shape how we take decisions (Eagly and Chaiken 1993; Damasio 1994; Lerner and Keltner 2000, 2001). Political psychologists have found that emotions can affect how willing we are to re-evaluate our political beliefs and become actively engaged in politics (Marcus and MacKuen 1993; Marcus et al. 2000; Rudolph et al. 2000; Brader 2005, 2006; Valentino et al. 2008, 2009; Huddy et al. 2007; Groenendyk 2011).

Some of this research has investigated the impact on political behavior of discrete emotions such as enthusiasm, anger, fear and sadness.^1^ For example, there is evidence that how we react to negative stimuli and threatening situations depends in part on whether they make us feel fear or anger (Huddy et al. 2005, 2007; Valentino et al. 2008; MacKuen et al. 2010). The reasons for this may lie in the different mental systems these emotions operate in (Marcus and MacKuen 1993; Cacioppo et al. 1999; MacKuen et al. 2010; Weber 2012). While fear makes us more risk-averse and vigilant (avoidance/surveillance systems), anger increases our readiness to address the threat directly while relying on previously learned routines (approach/disposition systems). For instance, some research has shown that it is anger rather than fear that leads citizens to engage in protest behavior by taking part in demonstrations or in other forms of political mobilization (van Zomeren et al. 2004; Leach et al. 2006; Smith et al. 2008).

Yet, we know far less about the *causes* of these discrete emotions (Brader et al. 2010). What is it about the threatening situation or the negative stimulus that leads some people to experience fear and others anger? Since these emotional reactions may be central to determining our further behavior, it is important to understand why these emotions arise in the first place. So far, political scientists and political psychologists have however not paid much attention to exploring the sources of discrete emotional reactions (though see Smith et al. 2008; Brader et al. 2010; and Petersen et al. 2012).

In this paper, I develop two hypotheses that build on cognitive appraisal theories of emotions, which suggest that the emotions we experience depend on our assessment of the situation (Smith and Ellsworth 1985; Lazarus 1991; Smith et al. 2008). First, it is likely that our perception of whether someone is responsible for a threat is a key determinant of whether we react to that threat with fear or anger. Specifically, anger is the more likely emotional reaction if the threat has an identifiable external cause.

Yet, blaming an actor on its own may not be enough to explain why someone reacts with anger instead of fear: instead, it may matter what kind of actor is blamed. My second hypothesis is therefore that anger is more likely than fear among individuals who believe that the actor responsible should be under their control and should care about their welfare. This means that reacting with anger should be more likely if there is an accountability relationship between the individual and the actor blamed (e.g. Smith and Ellsworth 1985; Lerner and Keltner 2000, 2001; Smith et al. 2008; Sell et al. 2009).

I test these hypotheses using emotional reactions to the financial crisis in the UK as measured in a 2005–2010 panel survey. The financial crisis is an event that presented an important economic threat to both individual citizens and the country as a whole. Both fear and anger are appropriate responses to the financial crisis: fear about the potential impact of the economic downturn, anger at the actions that made the crisis possible. Moreover, the survey evidence shows that people varied in their assignment of responsibility for the crisis. While some did assign blame, others were more uncertain about its causes or believed that the crisis ‘just happened’. Among those citizens who did hold specific actors responsible, there was also variation in who was blamed. Many blamed banks and financial institutions in the UK and abroad, but others also saw the national government, the European Union (EU) or other countries as responsible.

In this paper, I show that these variations help to account for why some British citizens were angry and others afraid in reaction to the financial crisis. Specifically, I present evidence that citizens who held an actor responsible for the crisis were more likely to be angry. In addition, citizens were particularly likely to be angry if they held the national government and (to a lesser extent) the EU responsible for the crisis. I argue that this is because citizens believe that these two actors should be under their control and should care about their welfare. In other words, anger is more likely to arise when an accountability relationship is perceived to be broken or violated. These findings are particularly robust given that the availability of panel data means that I can control tightly for emotional and partisan predispositions.

Whether and to whom voters assign blame for the economic situation is important in explaining emotional reactions. This finding significantly advances our understanding of the origins of emotions in politics. Moreover, anger and fear differ in their consequences: I thus show in a concluding analysis that experiencing anger (but not fear) led previous Labour voters to reconsider their vote choice in 2010. This is consistent with the theory that anger activates the approach rather than the avoidance system. Overall, this paper underlines the importance of principal-agent relationships and accountability in analyzing the role of the economy in political behavior. Perceptions of responsibility and control have been central to understanding the impact of economic perceptions on vote choice, and the evidence presented here provides further evidence of their importance in explaining how voters react to economic conditions (on perceptions of responsibility, see, e.g., Rudolph 2003; Hellwig et al. 2008; Marsh and Tilley 2010; Hellwig and Coffey 2011; Hobolt et al. 2013; on the extent of control, see, e.g., Powell and Whitten 1993; Anderson 2000, 2007; Johns 2011).

This paper is structured as follows. I begin by reviewing the differences between fear and anger as discrete emotional reactions and then consider how voters’ emotions may have been shaped by their assessment of responsibility for the financial crisis. I then describe the data, measurement approach and modeling strategy before presenting the results. I conclude by highlighting implications of my findings for understanding emotional reactions to politics in general.

## Fear and Anger as Discrete Emotional Responses

Much existing work on the role of voters’ emotions in politics has made use of the two-dimensional valence model of emotions (Marcus et al. 2000; Marcus 2003). This model suggests that positive and negative emotions are arranged on two separate and orthogonal dimensions. This means that positive and negative emotions can co-occur (Cassese and Weber 2011). Neuroscientific research has shown that these positive and negative emotions each affect decision-making (Damasio 1994; Marcus et al. 2000; Valentino et al. 2011; Cassese and Weber 2011). In particular, negative emotions lead to us to try to avoid harm and danger (‘avoidance’), while positive emotions foster reward-seeking and engagement (‘approach’; Gray 1990; Cacioppo et al. 1999; Huddy et al. 2007; Valentino et al. 2011).

In political science, the most prominent use of the two-dimensional model of emotions is as part of the affective intelligence theory (Marcus et al. 2000), which argues that emotions help to determine when we rely on heuristics and when we take decisions more cautiously (though see Ladd and Lenz 2008, 2011). Specifically, positive emotions such as enthusiasm activate the disposition system and reinforce existing behaviors and attitudes. In contrast, negative emotions such as anxiety activate the surveillance system and lead individuals to rely less on habit. Instead, negative emotions are argued to increase information-seeking and careful decision-making. In this approach, fear and anger have generally been treated as part of one underlying dimension containing negative emotions (Marcus and MacKuen 1993; Marcus et al. 2000; Rudolph et al. 2000).^2^

However, more recently the two-dimensional valence model has been criticized as overly simplistic (e.g. Petersen 2010; Huddy et al. 2007; Valentino et al. 2008, 2011; Smith et al. 2008; Weber 2012; see also Conover and Feldman 1986). Researchers have asked whether a focus on positive and negative emotions fails to account for differences between emotions of the same valence (Cassese and Weber 2011). In particular, it has been questioned whether anger and fear should both be seen as similar, negative emotions. Rather, it is argued that these two emotions should be considered as discrete responses because they are caused by different situational appraisals and because they have different consequences for our behavior (Smith and Ellsworth 1985; Conover and Feldman 1986; Lerner and Keltner 2000, 2001; Huddy et al. 2007; Carver and Harmon-Jones 2009; Petersen 2010; Valentino et al. 2011; Marcus 2013, p. 119).

First, fear and anger may lead us to engage in different kinds of further behavior. While most versions of affective intelligence theory argue that both emotions trigger the surveillance system (Marcus et al. 2000), more recent research indicates that the effects of fear and anger may differ. There is thus some evidence that anxiety leads to problem-focused information-seeking, risk-averse behavior and increased vigilance, while anger increases risk-seeking behavior and the motivation to engage in political action (Lerner and Keltner 2000, 2001; Druckman and McDermott 2008; Carver and Harmon-Jones 2009; Valentino et al. 2009, 2011; Brader et al. 2010; see also MacKuen et al. 2010 for a similar account using AIT theory). As Smith et al. (2008, p. 223) state, ‘anger motivates people to attack and remove the source of action,… and fear motivates people to be cautious and avoid harm’.^3^

Fear and anger also differ in how they are caused. The main insights into the different origins of fear and anger come from cognitive appraisal theories of emotions (Smith and Ellsworth 1985; Lazarus 1991; Smith et al. 2008; Carver and Harmon-Jones 2009; Cassese and Weber 2011; Valentino et al. 2011). These theories suggest that our emotional responses arise as a consequence of how we understand and interpret the situation we experience. As Best and Krueger (2011, p. 89) note: ‘Because people often appraise the same situation differently, cognitive appraisal theory helps comprehend why people experiencing the same phenomena or event may exhibit different emotions of the same valence.’ The focus of such theories is therefore on the cognitive appraisals that generate specific emotions.^4^ Whether a threat leads to fear or to anger therefore depends on how a threat is perceived and assessed. In the next section, I will consider how these appraisals may have shaped emotional reactions to the financial crisis.

## Fear and Anger as Emotional Responses to the Financial Crisis

The financial crisis since 2007 is likely to have been seen as both a personal and national threat to many people. As a negative stimulus, it will have led to strong emotional responses (‘affective appraisals’), including anger and fear (MacKuen et al. 2010). However, when did the financial crisis cause anger and when did it inspire fear? In line with discrete models of emotion, it is likely that the nature of individuals’ appraisal of the situation was an important determinant of their emotional reactions. Appraisal theories identify a wide variety of important factors, including among others the certainty and legitimacy of the outcome and the relevance of the event (Smith and Ellsworth 1985; Cassese and Weber 2011).

### How does Assigning Responsibility Affect Emotional Reactions?

Concerning the financial crisis, one aspect should be particularly important: the assignment of responsibility. Our emotional response to a threat can depend on whether we attribute blame for that threat to an actor, that is, whether we believe that another actor is responsible for the negative stimulus (Lerner and Tiedens 2006).^5^ In such cases, anger is the likely emotional response (Weiner 1985; Smith and Ellsworth 1985; Conover and Feldman 1986; Frijda 1986; Lazarus and Lazarus 1994; Berkowitz and Harmon-Jones 2004; Lerner and Tiedens 2006; Smith et al. 2008). Lerner and Keltner (2000, p. 479) thus note that individuals react with anger when they think that the situation was ‘brought about by others’ and is ‘under human control’ (see also Brader et al. 2010; Petersen et al. 2012). Smith and Ellsworth (1985) term these aspects of situation ‘other-control’ and ‘other-responsibility’. Thus, anger is caused by events that are the result of actions by an external actor, not by one’s own actions (‘self-control’) or simply the result of fate, luck or circumstance (‘situational control’).^6^

In contrast, fear is the more likely reaction when the situation cannot be blamed on any specific actor (Lerner and Keltner 2000; though see Smith et al. 2008). As Lerner and Tiedens (2006, p. 117) write, ‘when people feel uncertain or lack confidence about the cause of negative events, they are likely to feel fear and anxiety rather than anger.’^7^ In other words, fear can be seen as a ‘default’ response to a threat that is maintained if no external source for that threat can be identified.

We know that voters vary in whether they assign responsibility for economic outcomes to various actors. While certainly not an easy task, citizens can and do regularly identify actors responsible for economic developments (e.g., Powell and Whitten 1993; Anderson 2000, 2007; Rudolph and Grant 2002; Rudolph 2003; Marsh and Tilley 2010). The nature of the emotional response to the financial crisis should depend in part on whether an individual believed a specific actor was responsible. The first hypothesis is thus that *individuals should experience anger rather than fear if they blame an actor for the financial crisis*.

### Accountability Relationships and Anger

The expectation above is simple: assigning blame for a threat leads to increased anger compared to fear. Yet cognitive appraisal theories also indicate that the relationship between blame and emotional reactions may be more nuanced than this. It may matter what *kind* of actor we assign blame to, or more specifically whether there is a principal-agent relationship with that actor. Anger rather than fear should be more likely if this is the case. This is because individuals will be more likely to react with anger if they believe the threat to be caused by an external actor who (1) they should have control over but who (2) has paid insufficient regard to their welfare. I will address these two points in turn.

First, it has been established that anger is more likely if individuals themselves believe that they have some control over remedying the situation (Smith and Ellsworth 1985; Lazarus and Lazarus 1994; Lerner and Keltner 2001; Smith et al. 2008; Valentino et al. 2009). If they do believe this, then anger is a more likely reaction than fear. This appraisal is what Smith and Ellsworth (1985) refer to as ‘self-control’ and ‘self-responsibility’: the extent to which the individuals themselves believe that they have control over and responsibility for the events.^8^ Smith et al. (2008) note that other work uses similar terms such as control potential (Roseman et al. 1990), controllability (Frijda et al. 1989) or coping potential (Lazarus 2001). In political science, this has usually been termed political efficacy (Easton 1965; Easton and Dennis 1967; Balch 1974; Niemi et al. 1991).^9^ When the perception of efficacy is low, fear is the likely emotional reaction, while higher levels are associated with anger (Lerner and Keltner 2001). Thus, Valentino et al. (2009, p. 310) note that ‘[f]ear occurs when…the situation appears to be outside the individual’s control, while anger is more likely when the individual perceives herself to be in control of the causal agent.’ We are likely to react with anger when we think that we can control the external actor behind the threat, but with fear if addressing a threat by an actor of whom we are not the principal.

However, the key point that causes anger in politics may be that the agent over whom we nominally have control has not acted in ways that benefit us. Recent research in evolutionary psychology has argued that anger arises when ‘the other party is not placing ‘‘sufficient’’ weight on [our] welfare’ (Sell et al. 2009, p. 15074). This may particularly be the case when we have some control over the actor, since that leads us to expect benefits in return. This means that anger rather than fear may be the result of situations where we are disappointed about an actor who should be caring more about our welfare. In related work, anger has been seen to be caused when individuals believe an action to be illegitimate or improper (Berkowitz and Harmon-Jones 2004): ‘An angering event is one in which someone or something challenges what ‘ought’ to happen’ (Frijda 1986, p. 199; see also Petersen et al. 2012). Importantly, Roseman et al. (1996) argue that the perception of illegitimacy needs to be coupled with control potential to lead to anger. The question is not whether individuals have actual control over the external actor, but whether they believe they should have such control. In sum, angry emotional reactions may arise in particular when voters blame an actor with whom the principal-agent relationship is malfunctioning.

With regard to the financial crisis (and the economy in general), citizens can be said to be the principals of their national government, for example through elections (Müller 2000). At the very least, they are likely to hold the normative belief that these actors *should* be their agents and thus accountable to them. This institutionalized principal-agent relationship stands in contrast to the weak (or even non-existent) accountability ties that link citizens to other economic actors such as businesses, banks and foreign countries. Concerning the European Union, there is a weaker link between citizens and the EU’s political institutions. Nevertheless, though the chain of accountability is less direct, the EU is also under clearer citizen control than, say, banks or mortgage companies; at the very least, voters may expect the EU to be more responsive to citizen concerns.

Importantly, citizens are likely to have varied in whom they held responsible for the financial crisis. While some will have blamed banks and mortgage companies, others may have held governments responsible as well. In general, voters vary in who they believe has the most influence on economic outcomes. For example, they differ in they extent they see the government as responsible for the economy (Powell and Whitten 1993; Anderson 2000, 2007; Rudolph 2003). For example, in the 2005 BES pre-election survey, 25 % of respondents said that the government influences the performance of the economy ‘a great deal’, 54 % ‘a fair amount’, 11 % ‘not very much’ or ‘not at all’, with 9 % ticking ‘don’t know’.^10^ It is likely that this also applies to other actors with potential influence on the economy such as the European Union, banks, businesses and foreign countries and governments.

In sum, in the context of the financial crisis citizens should be angry rather than afraid if they blame the national government (and to a lesser extent the EU) for the threat, and they should be afraid rather than angry if they see the threat’s cause as associated with actors outside of clear principal-agent relationships such as banks or foreign governments.^11^ My second hypothesis is therefore that *individuals should be more likely to experience anger rather than fear if they blame the national government or the EU for the financial crisis than if they blame other actors.*

## Measuring Fear and Anger Using the BES Internet Panel

I study voters’ emotional reactions to the financial crisis in Great Britain using data from a 6-year internet panel survey carried out by the British Election Study (2010). This survey had nine waves, three each in the two election years (2005 and 2010) and one each in 2006, 2008 and 2009. The waves used in this paper are shown in Table 1. Sanders et al. (2007, 2011) show that the sample used by the BES internet panel is very similar to that of a representative in-person sample. Identical models of vote choice run with the in-person and internet surveys also yield almost identical model parameters (Sanders et al. 2007).

**Table 1:** BES internet panel

Wave	Date	Respondents	Questions
1	March/April 2005	7793	Emotional predispositions
3	May 2005	5910	Vote choice
4	May 2006	6,186	Attention to politics Internal efficacy Party identification Party affect (like-dislike) Economic ideology
6	July 2009	4,048	Assignment of responsibility
7	March/April 2010	3,402	Emotional reactions
9	May 2010	2,781	Vote choice

I construct my measure of emotional reactions to the financial crisis based on a question asked in the pre-election survey 2010 (wave 7). This survey asked two questions about respondents’ feelings about the economy: ‘Which, if any, of the following words describe your feelings about the country’s general economic situation?’ and ‘Which, if any, of the following words describe your feelings about how you have been personally affected by the current financial crisis?’. The respondents were given eight emotions to choose from: angry, happy, disgusted, hopeful, uneasy, confident, afraid and proud. They could also select ‘no feelings’ or ‘don’t know’. Of these eight emotions, respondents were allowed to choose up to four. I treat ‘angry’ as the indicator of anger and ‘afraid’ and ‘uneasy’ as indicators of fear.

My main dependent variable is the proportion of ‘anger’ relative to the total number of angry and fearful emotions selected by the respondent.^12^ This variable ranges from 0 to 1. Around 8 % of respondents selected neither angry nor fearful emotions. These respondents were coded as having a proportion of 0 angry emotions, but the statistical results do not change if this group of respondents is omitted (see supplemental material). In the analysis below, I do not take into account the total number of angry and fearful emotions selected; additional analyses presented in the supplemental materials show that the substance of the findings does not differ depending on the total number of these emotions selected by the respondents.

**Fig. 1:**
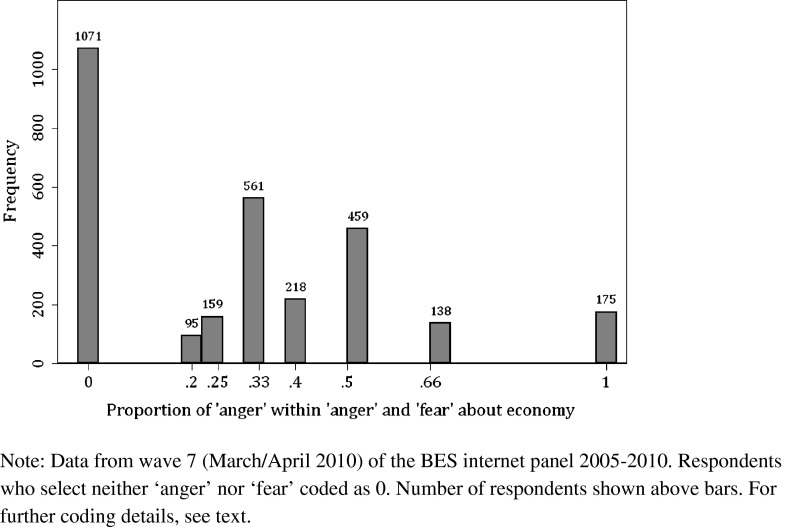
Emotional reactions to the financial crisis, 2010

Figure 1 presents a histogram of angry and fearful emotional reactions to the economy and the financial crisis in Britain in 2010. A little more than a third of respondents do not select anger at all. For about a quarter of respondents, anger makes up 50 % or more of the angry and fearful emotions selected. The remaining group of respondents, making up more than a third of the total, can be classified as slightly angry: while they do select that emotion, they do so less often than fearful emotions.

## Measures of Blame Assignment and Control Variables

Before analyzing the causes of anger and fear as emotional reactions to the financial crisis, I describe the model set-up, including the additional controls needed. First, the key predictor variables of fear and anger concern the responsibility of other actors for the financial crisis. One key advantage of using panel data is that I can measure perceptions of responsibility and emotional reactions in separate waves. These are taken from an earlier wave than the emotion measure in order to reduce the problem known as simultaneity in survey response, i.e. that survey participants try to remain consistent in their responses within one survey (Abelson 1968; Schwarz and Sudman 1992). Specifically, I use a question from wave 6 (July 2009) that measures blame assignment directly. Respondents were asked: ‘Who do you think is responsible for the current financial crisis?’ and were then asked to choose as many actors as they wished out of a list of eleven options. The answers ‘Gordon Brown’ and ‘the British government’ were coded as blaming the Labour government. Further indicators for responsibility are (1) international financiers and banks (both British and American), (2) American actors (George W. Bush, Barack Obama and the American government), (3) the European Union, (4) people with big mortgages or (5) no one, also including ‘don’t knows’.

The responses to this question in 2009 are shown in Table 2. As multiple selections were possible, the column entries add up to more than 100 %. Almost all respondents (86.6 %) hold national or international banks responsible for the crisis. About half of respondents believe that the US and British governments hold some responsibility, while only 20 % see the EU as responsible for the crisis. (Recall that this survey was carried out in July 2009, so before the crisis began to center on Greece, Italy and Spain.) 9 % of respondents selected ‘don’t know’ or ‘no actor responsible’ as their reply to this question.

**Table 2:** Assignments of responsibility for the financial crisis, 2009

Actors responsible	*n*	in %
Banks	3,499	86.6
National government	1,929	47.8
US government	1,791	44.3
Mortgage holders	1,142	28.3
European Union	818	20.3
Don’t know/no one	365	9.0
Total	4,040	

*Note* Multiple answers possible. Data from wave 6 (July 2009) of the BES internet panel 2005–2010. Unweighted results shown. For coding details, see text

I have suggested that blaming the national government and the EU leads to anger more than blaming other actors due to the existence of a principal-agent relationship, which in turn creates the possibility that individuals will believe that they have coping potential and that the actor blamed has acted improperly in neglecting their welfare. Unfortunately, the presence of these two perceptions (coping potential and disregard for welfare) cannot be tested directly using the data available. For one, the survey does not include measures that assess whether respondents think that governments should be responsive (rather than whether they are responsive). I do control for general efficacy using a measure from the 2006 wave that asked respondents to rate their influence on politics on a scale of 0–10.^13^ Yet this overall measure of efficacy does not capture whether respondents believe they *should* have influence over politics, and it is precisely this frustration and discrepancy that should lead to anger (Berkowitz and Harmon-Jones 2004). In addition, disregard for welfare is not measured either: there are no items that ask whether governments have a duty to care for their citizens or whether this duty has been violated during the crisis.

In the model, I also control for further factors that may be important influences on both emotional reactions to the financial crisis and on assignments of responsibility. Another advantage of the panel data is that I can measure these important confounders before the crisis occurred. First, I control for emotional predispositions towards anger and fear. To do so, I include a variable from wave 1 (March/April 2005) that measures the proportion of anger about the economy relative to the total number of angry and fearful emotions. This variable has three possible values: 0, 0.5 and 1. Respondents who were neither angry nor fearful are coded as 0. I also include a further variable from wave 1 that measures the proportion of anger about two unrelated matters, namely the National Health Service and the Iraq war. This index also ranges from 0 to 1 and has eight possible values. Overall, these two variables control for any factors that generally influence why people say they are angry rather than fearful about the economy or general political topics.

I also control for partisan affect. It is very important to control tightly for this given its strong influence on emotional reactions (Ladd and Lenz 2008, 2011), on perceptions of the economy (e.g. Gerber and Huber 2010) and on survey response overall (Rahn et al. 1994). I control for partisan affect by including indicators for party identification with the three main parties and for general affect for them, as measured in the last pre-crisis wave. Party identification is thus coded 1 for Labour, Conservative and Liberal Democrat identifiers respectively in wave 4 (May 2006), 0 otherwise.^14^ Those with a different or no party identification are used as the reference category. General affect is measured using the 0–10 like-dislike score for the three parties, also from wave 4 (May 2006), with higher values indicating more positive evaluations.^15^

I also control for economic ideology, measured as respondents’ preferences on cutting taxes or spending more on public services on a 0–10 scale. Left-wing respondents may be more likely to be angry about the crisis: ideologically, they may react more negatively towards financial institutions than right-wing voters. Since left–right economic ideology may also color opinions on responsibility for and handling of the crisis (Anderson 2007), it is important to control for this factor.^16^ Finally, I control for attention to politics in 2006 as panel attrition might be higher among respondents with lower levels of political interest.^17^

## Results

I model the dependent variable using ordinary least squares (OLS) regression; results using ordinal logistic regression lead to substantively identical results (see supplemental information). I present eight models (Tables 3, 4). The first four test the first hypothesis, so whether responsibility attributions increase anger relative to fear, first without control variables (Model 1) and then with different sets of attitudinal controls (Models 2–4). The second four models test the second hypothesis, so whether anger is more likely if the national government is blamed rather than other external actors, again without (Model 5) and with the various controls (Models 6–8). Below, I discuss results mainly from Models 3 and 7, which include the main attitudinal and partisan controls, but the results are consistent across models.

**Table 3:** Assignment of responsibility and emotional reactions (1)

	Model 1	Model 2	Model 3	Model 4
	No controls	Minimal controls	Controls for party ID	Controls for party affect
Actors responsible, 2009				
Actor blamed (*Ref: no one/DK*)	0.102*** (0.024)	0.074** (0.026)	0.072** (0.026)	0.070** (0.027)
Emotional predispositions, 2005				
Proportion of anger, economy		0.167*** (0.031)	0.140*** (0.031)	0.107*** (0.032)
Proportion of anger, NHS and Iraq		0.140*** (0.020)	0.122*** (0.020)	0.093*** (0.020)
Left–right economic ideology, 2006		0.019*** (0.003)	0.013*** (0.003)	0.010*** (0.003)
Attention to politics, 2006		0.010*** (0.003)	0.011*** (0.003)	0.009** (0.003)
Political efficacy, 2006		−0.007** (0.003)	−0.006* (0.003)	−0.004 (0.003)
Party identification, 2006				
Labour			−0.075*** (0.014)	−0.022 (0.017)
Conservative			0.025 (0.015)	−0.004 (0.018)
Liberal democrat			−0.041* (0.020)	−0.025 (0.021)
Party affect, 2006				
Labour				−0.015*** (0.003)
Conservative				0.003 (0.003)
Liberal democrat				−0.003 (0.003)
Constant	0.192*** (0.023)	0.029 (0.030)	0.085** (0.031)	0.162*** (0.038)
Observations	2,530	2,298	2,298	2,241
*R* ^2^	0.007	0.087	0.105	0.119
Adjusted *R* ^2^	0.007	0.084	0.102	0.114

*Note* The dependent variable is the proportion of ‘anger’ relative to the total number of angry and fearful emotions selected by the respondent (range: 0–1). Respondents selecting neither angry nor fearful emotions coded as 0. Standard errors in parentheses. * *p* < 0.05, ** *p* < 0.01, *** *p* < 0.001. Data from BES internet panel 2005–2010. For coding details of the predictor variables, see text

**Table 4:** Assignment of responsibility and emotional reactions (2)

	Model 5	Model 6	Model 7	Model 8
	No controls	Minimal controls	Controls for party ID	Controls for party affect
Actors responsible, 2009				
National government	0.146*** (0.013)	0.105*** (0.014)	0.089*** (0.014)	0.074*** 0.015)
United States	−0.018 (0.012)	−0.007 (0.013)	−0.005 (0.013)	−0.003 (0.013)
Banks	−0.073** (0.028)	−0.034 (0.029)	−0.028 (0.029)	−0.029 (0.030)
Mortgage holders	−0.002 (0.012)	−0.002 (0.012)	−0.004 (0.012)	−0.001 (0.012)
European Union	0.032* (0.014)	0.036* (0.015)	0.037* (0.015)	0.039** (0.015)
Don’t know/no one	−0.094** (0.036)	−0.051 (0.039)	−0.052 (0.038)	−0.059 (0.039)
Emotional predispositions, 2005				
Proportion of anger, economy		0.121*** (0.031)	0.109*** (0.031)	0.090** (0.031)
Proportion of anger, NHS and Iraq		0.122*** (0.020)	0.112*** (0.020)	0.092*** (0.020)
Left–right economic ideology, 2006		0.014*** (0.003)	0.011*** (0.003)	0.009** (0.003)
Attention to politics, 2006		0.008** (0.003)	0.009** (0.003)	0.008** (0.003)
Political efficacy, 2006		−0.005 (0.003)	−0.005 (0.003)	−0.003 (0.003)
Party identification, 2006				
Labour			−0.050*** (0.015)	−0.017 (0.017)
Conservative			0.016 (0.015)	−0.001 (0.018)
Liberal democrat			−0.026 (0.019)	−0.016 (0.021)
Party affect, 2006				
Labour				−0.010*** (0.003)
Conservative				0.001 (0.003)
Liberal democrat				−0.002 (0.003)
Constant	0.286*** (0.028)	0.115** (0.037)	0.148*** (0.038)	0.206*** (0.044)
Observations	2,530	2,298	2,298	2,241
*R* ^2^	0.080	0.126	0.133	0.140
Adjusted *R* ^2^	0.078	0.122	0.128	0.133

*Note* The dependent variable is the proportion of ‘anger’ relative to the total number of angry and fearful emotions selected by the respondent (range: 0–1). Respondents selecting neither angry nor fearful emotions coded as 0. Standard errors in parentheses. * *p* < 0.05, ** *p* < 0.01, *** *p* < 0.001. Data from BES internet panel 2005–2010. For coding details of the predictor variables, see text

Before we turn to the results relevant to the two hypotheses, I briefly describe the effects of the control variables, which mostly have the expected effects and are consistent across models. Thus, emotional predispositions are very influential: those voters who felt angrier about either the economy or Iraq and the NHS in 2005 are also more likely to feel angry about the financial crisis in 2010. Party identification with Labour reduces anger. The same pattern also holds if we control for general party affect (Model 4). These results are consistent with a partisan impact on emotional reactions (Ladd and Lenz 2008, 2011): since ‘their’ government was in charge, Labour supporters may have been less likely to experience anger. The more right-wing voters are in terms of economic ideology, the more likely they are to feel angry about the financial crisis; this goes against the prediction based on ideology, but fits with the impact of party affect. It may be that this item captures opposition to the fact that the crisis led to increased budget deficits and tax increases without cutting social spending. Finally, higher levels of attention to politics increase the probability of feeling angry compared to feeling afraid, while political efficacy has a weak but significant effect on the nature of negative emotional reactions.^18^

I begin with the first hypothesis, which states that citizens are more likely to feel anger if they blame an external actor. The key variable here is whether respondents chose one or more of the actors on the list of potential actors responsible; in other words, they did not answer ‘no one’ or ‘don’t know’. Models 1–4 show that people who blamed an actor were more likely to be angry compared to those respondents who did not.^19^ The increase in the proportion of anger relative to all anger/fear mentions is about 0.07. The effect is significant at the 0.05 level in all models. The effect is also of very similar magnitude to that of identifying with Labour and larger than that of economic ideology and attention to politics. There is thus support for the hypothesis that a substantively important predictor of anger about the crisis is whether an actor was seen as responsible for it.

The second hypothesis argued that the effect of blame assignment may be more nuanced, in that it matters *whom* citizens assign blame to. This hypothesis is tested in Models 5–8 (Table 4), where our main attention should be on the different responsibility indicators.

The results provide strong support for the hypothesis. Voters who believe that the UK government was one of the actors responsible for the financial crisis are more likely to be angry. The proportion of anger relative to all anger/fear mentions increases by 0.09 on the 0–1 scale. This effect is significant at the 0.001 level and substantively large compared to those of party identification, ideology and attention to politics. Blaming the EU also increases the proportion of anger relative to all anger/fear mentions; at 0.04 (*p* < 0.05), this effect is smaller than that of blaming the national government (Wald test, *p* < 0.05). The coefficient for blaming banks is negative but only significant in models without controls.

In sum, holding the UK government responsible has a large effect on the proportion of anger within anger and fear, while blaming the EU has a smaller, but still significant effect. Together with the fact that blaming banks has no significant effect, this pattern of results indicates that an accountability relationship appears to be necessary for blame to result in anger. Finally, the reasons why blaming banks and not blaming an actor at all fail to lead to anger are different: in the former case, the reason is a lack of an accountability relationship, while in the latter case the reason is the failure to identify an external cause for the threat.

## The Consequences of Emotional Reactions

Anger should activate the approach system and lead individuals to attempt to remove the source of harm, while fear should activate the avoidance system and lead to risk-averse behavior. This expectation can be applied to vote choice after the financial crisis. The default decision of British citizens who voted Labour in 2005 might be to vote Labour again in 2010. However, anger about the financial crisis might lead such voters to re-evaluate their standing decision and try to remove Labour government, i.e. the source of the threat. In contrast, fear might lead voters to stick with their standing decision.^20^ So, I expect Labour voters in 2005 who are angry about the financial crisis to be less likely to vote Labour again in 2010. In contrast, Labour voters in 2010 who are fearful about the crisis should be likely to vote Labour again.

I test this using a binary logistic regression model (Table 5). The sample in Models 9 and 10 is restricted to Labour voters in 2005 who also voted in 2010. The dependent variable is measured as 1 if the respondent voted for Labour again in 2010, 0 if he/she voted for another party. Anger is measured as the number of times the respondent selected ‘angry’ as an emotion regarding the economy and the financial crisis; this variable ranges from 0 to 2. Fear is measured as the number of times the respondent selected ‘afraid’ and ‘uneasy’ as an emotion regarding the economy and the financial crisis; this variable ranges from 0 to 4. Model 9 shows the results of a simple model, while Model 10 adds key controls for emotional predispositions, partisan leanings and economic evaluations (see supplemental information for coding details).

**Table 5:** Emotional reactions and vote choice

	Model 9	Model 10	Model 11	Model 12
	2005 labour voters only		All 2005 voters	
	No controls	Controls included	No controls	Controls included
Anger mentions, 2010 (0–2)	−0.410** (0.129)	−0.344* (0.155)	0.063 (0.080)	−0.013 (0.108)
Fear mentions, 2010 (0–4)	−0.193* (0.082)	−0.074 (0.101)	−0.032 (0.054)	−0.051 (0.069)
Voted labour in 2005			0.298 (0.213)	−0.612* (0.266)
Voted labour * Anger mentions			−0.474** (0.152)	−0.378* (0.173)
Voted labour * Fear mentions			−0.162 (0.098)	−0.074 (0.113)
Identified with party of 2005 vote, 2006		1.216*** (0.300)		1.166*** (0.145)
Like-dislike for party of 2005 vote, 2006		0.189** (0.062)		0.182*** (0.035)
Anger mentions, NHS and Iraq, 2005 (0–2)		0.355 (0.194)		0.170 (0.100)
Fear mentions, NHS and Iraq, 2005 (0–4)		−0.027 (0.111)		−0.009 (0.061)
Anger mention, economy, 2005 (0/1)		1.034 (0.588)		−0.196 (0.211)
Fear mentions, economy, 2005 (0–2)		−0.470 (0.279)		−0.171 (0.149)
Personally affected by crisis, 2010 (0–3)		0.357* (0.180)		0.186 (0.102)
Egocentric economic evaluations, 2010		−0.141 (0.182)		−0.169 (0.106)
Sociotropic economic evaluations, 2010		−0.320* (0.151)		0.049 (0.089)
Constant	1.081*** (0.161)	−0.969 (0.617)	0.782*** (0.139)	−0.568 (0.358)
Observations	516	476	1,678	1,425

*Note* The dependent variable is 1 if the respondent voted for the same party in 2005 and 2010, 0 if not. Standard errors in parentheses. * *p* < 0.05, ** *p* < 0.01, *** *p* < 0.001. Data from BES internet panel 2005–2010. For coding details of the predictor variables, see text

Based on Model 9, we can calculate that previous Labour supporters were about 14 % less likely to vote for Labour in 2010 if they were very angry about the economy rather than not angry at all.^21^ This effect is significant at the .05 level, and it is also substantively large: to compare, the effect of identifying with Labour in 2005 increases the probability of voting for Labour again in 2010 by 27 %.

Models 11 and 12 show that the effect of anger was restricted to Labour voters. These models use the full sample of 2005 voters; the dependent variable is 1 if the respondent voted for the same party as in 2005, 0 if not. Emotional reactions are interacted with having voted Labour in 2005. The negative statistical significance of the interaction term along with the absence of an effect among other voters shows that anger led to a change in behavior specifically among previous Labour voters.

## Discussion and Conclusion

When are voters angry in reaction to the economic situation, and when are they afraid? In this paper, I have argued that citizens respond with anger when they hold an external actor responsible for the crisis. Moreover, anger is particularly likely when individuals see the actor responsible as their agent. Individuals are more likely to get angry if they think the threat arose due to the actions of an agent who should have placed greater weight on their welfare. These two hypotheses held in the case of the financial crisis: voters were angrier when they assigned blame for the crisis, but anger was increased only when they blamed the national government and, to a lesser extent, the EU.

These findings are important because understanding how citizens vary in how they interpret and appraise negative events helps us explain why their political reactions to these events differ (MacKuen et al. 2010). There has been a lot of recent work examining how variation in emotional reactions leads to differences in political behavior, for example in terms of vote choice, political attitudes, participation and learning (Weber 2012). However, a full picture of the importance of emotions in politics also requires an understanding of the determinants of these emotional reactions. This paper shows that the nature of blame assignment has a strong influence on emotional reactions.

Finally, it is important to note important limitations of the present findings; these also present potential ways of extending this research. First, it was not possible to test the specific causal mechanism why individuals were angry about the economy when they blamed their political agents. It was suggested that the likely cause is a perception that the actor should be under the individual’s control but that has paid insufficient attention to the individual’s welfare. Due to the lack of suitable survey measures in the British Election Study, the accuracy of this suggested mechanism cannot be definitively confirmed here.

Second, this paper has argued that the causal arrow between anger and blame is in the following direction: a threat to an individual arises; the individual decides whether or not to assign blame for that threat to an actor; this appraisal of the situation leads the individual to experience certain emotions, including anger and fear. However, it may also be that angry individuals seek to assign blame (Frijda 1993; Berkowitz and Harmon-Jones 2004), and that such people may decide to blame in particular those actors whose principal they are. Existing research in psychology has not been able to disentangle these two plausible causal stories, and providing a clearer causal account should be a task for future research.

Third, this research has highlighted the importance of distinguishing between fear and anger. However, other negative emotions exist as well. In particular, disgust has been shown to have political importance (e.g. Vandenbroek 2011; Banks and Valentino 2012), and its origins also deserve to be studied.

Finally, future work should engage in a comparative effort. Blame assignments are strongly shaped by institutional arrangements. For example, voters may be more willing to attribute responsibility for economic outcomes to the government where there is ‘clarity of responsibility’ (Powell and Whitten 1993; Anderson 2000), both at the national level and across other levels of governance. Whether there is clarity of responsibility depends partly on the institutional structure of the state; for example, electoral systems affect the existence of clear alternatives in a party system (Powell and Vanberg 2000). This may also mean that anger in reaction to negative economic events is also greater in systems with higher clarity of responsibility. As a result, how emotional reactions to political events differ across countries may depend on quite familiar concepts and institutions related to citizen control. These emotions may then play an important role in determining how the economy influences political behavior.

## Electronic supplementary material

Below is the link to the electronic supplementary material.

Supplementary material 1 (DOC 121 kb)

## Footnotes

1 In this paper, fear and anxiety are treated as synonymous terms.

2 One important exception is MacKuen et al. (2010), who argue that aversion, which includes anger and disgust, activates the disposition system, just like positive emotions.

3 In their summary of recent research on the consequences of fear and anger, Valentino et al. (2008, p. 249) however note that while the finding that anger mobilizes action is clear and strong, the consequences of fear may be more variable.

4 This fits with the way political science researchers measure emotional responses, namely by asking participants in surveys and experiments to report their emotional reactions (Valentino et al. 2011). This means that the emotions political scientists generally tend to study are emotions that are consciously experienced and are reported after some level of individual self-examination.

5 It is worth noting that the link between blame and anger is not undisputed. For example, Berkowitz and Harmon-Jones (2004, p. 117) write that ‘[t]he anger evoked by the aversive state of affairs could at times seem unreasonable to those affected and thereby provoke a search for some external source to blame,… perhaps in an attempt to justify the feelings, ideas, and impulses that are experienced.’ A similar argument is made by Frijda (1993). At the very least, there may be a reciprocal relationship between blame and anger.

6 In a different context and based on evolutionary psychology, Petersen et al. (2012) show that anger towards welfare recipients depends on whether they are seen as responsible for their own poverty, e.g. because they lack motivation to work.

7 They add that sadness is caused when we blame general ‘situational’ events for the threat and shame/guilt when we blame ourselves for the event.

8 See Berkowitz and Harmon-Jones (2004, p. 116) for arguments against the importance of coping potential as a cause of anger.

9 The concept of self-control does not clearly relate to either internal or external efficacy. Internal efficacy is the extent to which an individual considers herself to be competent to understand and participate in politics, external efficacy the extent an individual considers the government to be responsive to citizen demands (Niemi et al. 1991). A perception of control over actors arguably requires both types of efficacy.

10 Weighted using YouGov’s non-political weight. The distribution of responses is similar if we use the same question asked in the 2010 survey and if we use the 2005 question asking about government influence on one’s personal financial situation.

11 Unfortunately, the BES questionnaire does not contain items that measure the extent to which individuals see situational causes as responsible for the crisis (e.g. globalization, capitalism or greed). Cognitive appraisal theories would predict that perceptions of situational control should lead to increased fear relative to anger.

12 ‘Disgust’, the final negative emotion that respondents could select, is left out of the analysis. While potentially related to anger (Marcus et al. 2000; MacKuen et al. 2010), many researchers now separate these two emotions (e.g. Vandenbroek 2011; Banks and Valentino 2012).

13 The question text is: ‘On a scale from 0 to 10, where 10 means a great deal of influence and 0 means no influence, how much influence do *you* have on politics and public affairs?’.

14 Wave 4 answers used because the party identification questions were not asked in the standard format in wave 1. The question text from wave 4 is: ‘Generally speaking, do you think of yourself as Labour, Conservative, Liberal Democrat or what?’.

15 The question text is: ‘On a scale that runs from 0 to 10, where 0 means strongly dislike and 10 means strongly like, how to you feel about the Labour Party?’.

16 0 means that ‘government should cut taxes a lot and spend much less on health and social services’, and 10 that ‘government should raise taxes a lot and spend much more on health and social services’. The coding of this variable was reversed in the analysis.

17 The question text is: ‘On a scale of 0–10, how much attention do you generally pay to politics?’.

18 This weak effect can be explained by the decision to include respondents without any emotion in the model; models excluding these respondents show no significant effect of political efficacy (see supplemental information).

19 Note that respondents expressing neither anger nor fear are coded as 0. The results do not change substantively if these respondents are excluded (see supplemental material).

20 Looking at turnout is not possible using the panel data due to the very low number of respondents reporting abstention in 2010.

21 These predicted probabilities are calculated using the observed-values approach suggested in Hanmer and Ozan Kalkan (2012).
