# Supplementary material for: Fear and Anger in Great Britain: Blame Assignment and Emotional Reactions to the Financial Crisis
Source: Polit Behav. 2013 Jul 9;36(3):683–703. doi: 10.1007/s11109-013-9241-5 (PMC4734452; doi:10.1007/s11109-013-9241-5)
Supplement: Supplementary file 1 — Supplementary material 1 (DOC 121 kb) [file 11109_2013_9241_MOESM1_ESM.doc]

**Supplemental information**

This document presents supplemental information on the manuscript ‘Fear and anger in Great Britain: blame assignment and emotional reactions to the financial crisis’.

1. *Dependent variable: distribution by total number of angry and fearful emotions*

The table below presents the distribution of the proportion of anger relative to the total number of anger and fearful emotions selected in wave 7 of the BES survey (March/April 2010). The columns show the number of times ‘angry’ was chosen, the rows the number of times ‘angry’, ‘afraid’ and/or ‘uneasy’ were chosen.

| **No. of times 'angry', 'afraid' and/or 'uneasy' selected** | **No. of times 'angry' selected** | | |  |
| --- | --- | --- | --- | --- |
|  | 0 | 1 | 2 | Total |
| 0 | 238 |  |  | 238 |
| Row % | 100 |  |  |  |
| 1 | 333 | 93 |  | 426 |
| Row % | 78.17 | 21.83 |  |  |
| 2 | 339 | 179 | 82 | 600 |
| Row % | 56.5 | 29.83 | 13.67 |  |
| 3 | 111 | 252 | 138 | 501 |
| Row % | 22.16 | 50.3 | 27.54 |  |
| 4 | 50 | 159 | 280 | 489 |
| Row % | 10.22 | 32.52 | 57.26 |  |
| 5 |  | 95 | 218 | 313 |
| Row % |  | 30.35 | 69.65 |  |
| 6 |  |  | 309 | 309 |
| Row % |  |  | 100 |  |
| Total | 1,071 | 778 | 1,027 | 2,876 |
| Row % | 48.00 | 22.41 | 29.59 |  |

1. *Robustness check: Anger versus fear only*

The following three regressions re-run Models 1 to 8 but exclude respondents who experienced neither anger nor fear. These are coded as 0 in the Models included in the paper. Failing to blame an actor could indicate apathy rather than anxiety, so it is important to check whether the results depend on this coding decision. The results are consistent in terms of magnitude and significance, though Model A4 is marginally non-significant at the .05 level (but still statistically significant at the 0.1 level).

|  | **Model A1** | **Model A2** | **Model A3.1** | **Model A4** |
| --- | --- | --- | --- | --- |
|  | **No controls** | **Minimal controls** | **Controls for party ID** | **Controls for party affect** |
| **Actors responsible, 2009** |  |  |  |  |
| Actor blamed (*Ref: no one/DK*) | 0.091^***^ (0.026) | 0.060^*^ (0.029) | 0.059^*^ (0.028) | 0.057 (0.030) |
| **Emotional predispositions, 2005** |  |  |  |  |
| Proportion of anger, economy |  | 0.148^***^ (0.032) | 0.128^***^ (0.032) | 0.098^**^ (0.032) |
| Proportion of anger, NHS and Iraq |  | 0.111^***^ (0.020) | 0.101^***^ (0.021) | 0.074^***^ (0.021) |
| Left-right economic ideology, 2006 |  | 0.017^***^ (0.003) | 0.012^***^ (0.003) | 0.010^**^ (0.003) |
| Attention to politics, 2006 |  | 0.011^***^ (0.003) | 0.011^***^ (0.003) | 0.009^**^ (0.003) |
| Political efficacy, 2006 |  | -0.005 (0.003) | -0.005 (0.003) | -0.002 (0.003) |
| **Party identification, 2006** |  |  |  |  |
| Labour |  |  | -0.057^***^ (0.015) | -0.008 (0.018) |
| Conservative |  |  | 0.023 (0.015) | 0.002 (0.018) |
| Liberal Democrat |  |  | -0.040 (0.020) | -0.023 (0.022) |
| **Party affect, 2006** |  |  |  |  |
| Labour |  |  |  | -0.014^***^ (0.003) |
| Conservative |  |  |  | 0.001 (0.003) |
| Liberal Democrat |  |  |  | -0.003 (0.003) |
| Constant | 0.228^***^ (0.025) | 0.073^*^ (0.033) | 0.115^***^ (0.034) | 0.193^***^ (0.041) |
| Observations | 2322 | 2109 | 2109 | 2056 |
| *R*^2^ | 0.005 | 0.066 | 0.078 | 0.089 |
| Adjusted *R*^2^ | 0.005 | 0.063 | 0.074 | 0.083 |

Note: The dependent variable is the proportion of ‘anger’ relative to the total number of angry and fearful emotions selected by the respondent (range: 0 to 1). Respondents selecting neither angry nor fearful emotions excluded. Standard errors in parentheses. * p<0.05, ** p<0.01, *** p<0.001.

|  | **Model A5** | | **Model A6** | | **Model A7** | | **Model A8** | |
| --- | --- | --- | --- | --- | --- | --- | --- | --- |
|  | **No controls** | | **Minimal controls** | | **Controls for party ID** | | **Controls for party affect** | |
| **Actors responsible, 2009** | |  | |  | |  | |  |
| National government | 0.123^***^ (0.014) | | 0.090^***^ (0.015) | | 0.078^***^ (0.015) | | 0.066^***^ (0.016) | |
| United States | -0.017 (0.013) | | -0.008 (0.014) | | -0.006 (0.014) | | -0.005 (0.014) | |
| Banks | -0.077^**^ (0.029) | | -0.036 (0.030) | | -0.032 (0.030) | | -0.028 (0.031) | |
| Mortgage holders | -0.002 (0.012) | | -0.003 (0.013) | | -0.005 (0.013) | | -0.002 (0.013) | |
| European Union | 0.028 (0.015) | | 0.033^*^ (0.015) | | 0.033^*^ (0.015) | | 0.036^*^ (0.015) | |
| Don't know / no one | -0.096^*^ (0.038) | | -0.045 (0.041) | | -0.047 (0.041) | | -0.048 (0.042) | |
| **Emotional predispositions, 2005** | |  | |  | |  | |  |
| Proportion of anger, economy |  | | 0.112^***^ (0.032) | | 0.103^**^ (0.032) | | 0.085^**^ (0.032) | |
| Proportion of anger, NHS and Iraq |  | | 0.101^***^ (0.020) | | 0.095^***^ (0.020) | | 0.075^***^ (0.021) | |
| Left-right economic ideology, 2006 |  | | 0.013^***^ (0.003) | | 0.010^***^ (0.003) | | 0.009^**^ (0.003) | |
| Attention to politics, 2006 |  | | 0.009^**^ (0.003) | | 0.009^**^ (0.003) | | 0.008^**^ (0.003) | |
| Political efficacy, 2006 |  | | -0.003 (0.003) | | -0.003 (0.003) | | -0.002 (0.003) | |
| **Party identification, 2006** | |  | |  | |  | |  |
| Labour |  | |  | | -0.034^*^ (0.015) | | -0.004 (0.018) | |
| Conservative |  | |  | | 0.016 (0.015) | | 0.004 (0.018) | |
| Liberal Democrat |  | |  | | -0.025 (0.020) | | -0.015 (0.022) | |
| **Party affect, 2006** | |  | |  | |  | |  |
| Labour |  | |  | |  | | -0.010^***^ (0.003) | |
| Conservative |  | |  | |  | | -0.000 (0.003) | |
| Liberal Democrat |  | |  | |  | | -0.002 (0.003) | |
| Constant | 0.324^***^ (0.029) | | 0.148^***^ (0.039) | | 0.172^***^ (0.040) | | 0.228^***^ (0.046) | |
| Observations | 2322 | | 2109 | | 2109 | | 2056 | |
| *R*^2^ | 0.059 | | 0.096 | | 0.100 | | 0.105 | |
| Adjusted *R*^2^ | 0.056 | | 0.091 | | 0.094 | | 0.098 | |

Note: The dependent variable is the proportion of ‘anger’ relative to the total number of angry and fearful emotions selected by the respondent (range: 0 to 1). Respondents selecting neither angry nor fearful emotions excluded. Standard errors in parentheses. * p<0.05, ** p<0.01, *** p<0.001.

1. *Robustness check: Respondents grouped by total number of angry and fearful emotions*

The following three regressions re-run Model 7, but on two sub-samples: (a) those mentioning 1 or 2 negative emotions, (b) those mentioning between 3 and 5 negative emotions. (Respondents with 0 or 6 total negative emotions are not included as these do not vary in the proportion of angry emotions selected.)

|  | | **Model A7.1** | | **Model A7.2** | |  |
| --- | --- | --- | --- | --- | --- | --- |
|  | | **1 or 2 negative emotions** | | **3, 4, or 5 negative emotions** | |  |
| **Actors responsible, 2009** |  | |  | |  |  |
| National government | | 0.083^*^ (0.036) | | 0.064^***^ (0.014) | |  |
| United States | | 0.002 (0.032) | | -0.019 (0.012) | |  |
| Banks | | -0.054 (0.072) | | -0.041 (0.030) | |  |
| Mortgage holders | | -0.032 (0.029) | | 0.018 (0.012) | |  |
| European Union | | 0.033 (0.040) | | 0.047^***^ (0.013) | |  |
| Don't know / no one | | -0.116 (0.092) | | -0.012 (0.042) | |  |
| **Emotional predispositions, 2005** |  | |  | |  |  |
| Proportion of anger, economy | | 0.221^*^ (0.089) | | 0.084^**^ (0.028) | |  |
| Proportion of anger, NHS and Iraq | | 0.129^**^ (0.046) | | 0.069^***^ (0.019) | |  |
| **Party identification, 2006** |  | |  | |  |  |
| Labour | | -0.052 (0.033) | | -0.018 (0.015) | |  |
| Conservative | | 0.036 (0.040) | | 0.014 (0.014) | |  |
| Liberal Democrat | | -0.073 (0.045) | | 0.002 (0.019) | |  |
| Left-right economic ideology, 2006 | | 0.021^**^ (0.007) | | 0.004 (0.003) | |  |
| Attention to politics, 2006 | | 0.015^*^ (0.006) | | 0.010^***^ (0.003) | |  |
| Political efficacy, 2006 | | 0.002 (0.006) | | -0.009^***^ (0.003) | |  |
| Constant | | 0.074 (0.097) | | 0.251^***^ (0.037) | |  |
| Observations | | 806 | | 1058 | |  |
| *R*^2^ | | 0.111 | | 0.126 | |  |
| Adjusted *R*^2^ | | 0.095 | | 0.114 | |  |

Note: The dependent variable is the proportion of ‘anger’ relative to the total number of angry and fearful emotions selected by the respondent (range: 0 to 1). Respondents selecting neither angry nor fearful emotions coded as 0. Standard errors in parentheses. * p<0.05, ** p<0.01, *** p<0.001.

1. *Robustness check: Ordinal logistic regression*

The following two regressions re-run Models 3 and 7 using ordinal logistic regression. The results are consistent with the OLS regression presented in the main paper. For example, the direction and significance of the key coefficients are the same. Note that the cut points are not shown.

|  | **Model A3.2** | | **Model A7.3** | |  | | |
| --- | --- | --- | --- | --- | --- | --- | --- |
| **Actors responsible, 2009** |  | |  | |  | | |
| National government |  | | 0.746^***^ (0.103) | |  | | |
| United States |  | | 0.014 (0.091) | |  | | |
| Banks |  | | -0.177 (0.210) | |  | | |
| Mortgage holders |  | | 0.002 (0.084) | |  | | |
| European Union |  | | 0.258^**^ (0.099) | |  | | |
| Don’t know/No one |  | | -0.342 (0.283) | |  | | |
| Actor blamed | 0.621^**^ (0.193) | | -0.304 (0.293) | |  | | |
| **Emotional predispositions, 2005** | |  | |  | |  |  |
| Proportion of anger, economy | 1.008^***^ (0.203) | | 0.803^***^ (0.207) | |  | | |
| Proportion of anger, NHS and Iraq | 0.925^***^ (0.139) | | 0.868^***^ (0.140) | |  | | |
| **Party identification, 2006** | |  | |  | |  |  |
| Labour | -0.609^***^ (0.103) | | -0.406^***^ (0.105) | |  | | |
| Conservative | 0.211^*^ (0.100) | | 0.147 (0.101) | |  | | |
| Liberal Democrat | -0.261 (0.138) | | -0.144 (0.138) | |  | | |
| Left-right economic ideology, 2006 | 0.087^***^ (0.019) | | 0.075^***^ (0.019) | |  | | |
| Attention to politics, 2006 | 0.068^***^ (0.019) | | 0.051^**^ (0.019) | |  | | |
| Political efficacy, 2006 | -0.054^**^ (0.019) | | -0.044^*^ (0.019) | |  | | |
| Observations | 2109 | | 2109 | |  | | |

Note: The dependent variable is the proportion of ‘anger’ relative to the total number of angry and fearful emotions selected by the respondent (range: 0 to 1). Respondents selecting neither angry nor fearful emotions coded as 0. Standard errors in parentheses. * p<0.05, ** p<0.01, *** p<0.001.

1. *The political consequences of anger versus fear regarding the financial crisis: variable coding*

In Models 10 and 12 I control for party identification, party affect, emotional predispositions, economic evaluations and the personal impact of the financial crisis. The coding for these variables is as in Model 1 to 8, apart from economic evaluations and personal impact of the crisis, which are coded as follows:

- Economic evaluations: Two variables measuring (1) sociotropic (and (2) egocentric economic evaluations regarding the past and future 12 months; each variable is coded on a 0-4 scale and is an index of two items. Higher values equal more negative evaluations of the economy; measured in wave 7 (2010).
- Personal impact of the financial crisis: One variable measuring the extent to which the crisis personally affected the respondent, coded on a 0-3 scale. Higher values mean that the respondent was more affected by the crisis; measured in wave 7 (2010).
